# Patients’ experiences of diagnosis and management of papillary thyroid microcarcinoma: a qualitative study

**DOI:** 10.1186/s12885-018-4152-9

**Published:** 2018-03-02

**Authors:** Brooke Nickel, Juan P. Brito, Ray Moynihan, Alexandra Barratt, Susan Jordan, Kirsten McCaffery

**Affiliations:** 1 Wiser Healthcare, Sydney School of Public Health, The University of Sydney, Sydney, 2006 NSW Australia; 2 Centre for Medical Psychology and Evidence-based Decision-making (CeMPED), The University of Sydney, Sydney, 2006 NSW Australia; 3 Knowledge and Evaluation Research Unit, Division of Endocrinology, Diabetes, Metabolism, and Nutrition, Mayo Clinic, Rochester, MN 55905 USA; 4 Centre for Research in Evidence-Based Practice, Bond University, Robina, 4226 QLD Australia; 5 QIMR Berghofer Medical Research Institute, Brisbane City, QLD 4006 Australia; 6 School of Public Health, The University of Queensland, St Lucia, 4072 QLD Australia

**Keywords:** Papillary thyroid cancer, Communication, Decision making, Treatment preferences, Overdiagnosis, Overtreatment

## Abstract

**Background:**

In recent years management practices in relation to low-risk papillary microcarcinoma (PMC) have been evolving with increased awareness of the potential overdiagnosis and overtreatment of PMCs, and guidelines recommendations for non-surgical management options such as active surveillance. This study aimed to develop an in-depth understanding of patients’ experiences of the communication of their PMC diagnosis, their treatment preferences and decision making.

**Methods:**

Semi-structured qualitative interviews with 25 patients diagnosed pre-operatively with PMC < 1 year since their diagnosis and treatment. Interviews were conducted between September 2015 and July 2016 and were audio-recorded and transcribed verbatim. Framework analysis method was used to analyse the data.

**Results:**

The diagnosis and treatment experience of PMC patients varied widely. The majority of patients were asymptomatic, and their PMC was initially detected via an imaging test requested for a reason unrelated to a thyroid disorder or symptom. Clinicians generally described PMC to patients as being a “small” or “slow-growing” cancer, and there was little evidence that clinicians had discussions about the possibility of overdiagnosis or overtreatment. Overall, surgery was the only option discussed and offered to patients. Patients preference for treatment was largely based on eliminating the possibility of the cancer spreading (thyroidectomy) or not wanting to be on thyroid replacement medication for the rest of their life (hemi-thyroidectomy). Many patients reported emotional and physical side-effects associated with their diagnosis and treatment, however patients generally indicated that active surveillance is not something they would have been interested in if it was offered to them.

**Conclusions:**

Evidence continues to emerge that many patients with PMCs may be overdiagnosed, and management guidelines are recommending more conservative management options for these patients. As a result, shared decision making around treatment options is vital so that patients are fully aware of the meaning of their diagnosis and their management options including active surveillance. Importantly, interventions to reduce unnecessary diagnoses of PMC are critically needed.

**Electronic supplementary material:**

The online version of this article (10.1186/s12885-018-4152-9) contains supplementary material, which is available to authorized users.

## Background

Papillary thyroid cancer (PTC) accounts for approximately 90% of all thyroid malignancies. The recent increase in the incidence of PTC [1] is largely due to the surge in the diagnosis of small (< 1 cm) PTCs [2], also known as papillary microcarcinomas (PMCs). It is highly probable that the introduction and growing use of imaging techniques (e.g. computed tomography (CT), high resolution ultrasound) has increased the detection of PMCs from a substantial reservoir of thyroid cancer [3]. This has raised concerns about overdiagnosis and in turn the overtreatment of patients diagnosed with PMC [4, 5].

Patients with PMC are usually treated surgically with either complete (total thyroidectomy) or partial (hemi-thyroidectomy) removal of the thyroid, although active surveillance with surgery if disease progression occurs may be a safe management option. Two observational studies of active surveillance in patients with PMCs concluded that the rate of loco-regional metastases with active surveillance is comparable to the rate that can occur after thyroid surgery, and that outcomes of surgery are the same whether the surgery is undertaken immediately or after any progression (tumour growth ≥3 mm or loco-regional metastasis) [6–8]. Moreover, it has been showed that delayed surgery in patients initially following active surveillance is safe, and does not lead to worse outcomes than patients who elected immediate surgery [9].

In light of recent evidence of the safety of active surveillance to manage patients diagnosed with PMC, the 2015 American Thyroid Association (ATA) guidelines now suggest that an active surveillance management approach can be considered as an alternative to immediate surgery in patients considered to have very low-risk tumours [10]. Clinical practice may now be changing with some clinicians beginning to offer active surveillance for low-risk PMC [9, 11].

With increasing awareness of the potential overdiagnosis and overtreatment of PMCs, and recent guidelines recommendations for active surveillance, it is important to understand the diagnosis and treatment experience from the patient perspective. In particular, understanding the communication around diagnosis and management options (including active surveillance) as well as factors that affected decision making may help patients calibrate prognosis with decision making. In addition, we were interested to know how a change in cancer terminology might change patient’s perceptions of their diagnosis. To this end, we conducted the first qualitative study in patients with PMCs to develop an in-depth understanding of patients’ experiences of the communication of their PMC diagnosis, their treatment preferences and how their treatment decisions were made.

## Methods

### Study design

This qualitative study used semi-structured face-to-face and telephone interviews to explore PMC patient’s diagnostic and treatment experience including communication and decision making.

### Participants

Participants were 25 PMC patients from New South Wales and Queensland, Australia. Eligible patients were < 1 year since initial diagnosis and were only included if they had been diagnosed pre-operatively (before any surgical intervention) with a single focus < 1 cm PTC with no evidence of extra-thyroidal extension or lymph node metastasis. All patients were aged 18 years and above and spoke adequate English as interpreting services were not used in this study.

### Recruitment

Patients were purposively recruited through three different avenues which included: 1) the Queensland Thyroid Cancer Study (QTCS) run by the QIMR Berghofer Medical Research Institute in Brisbane, Queensland; 2) patient clinics at the Chris O’Brien Lifehouse in Sydney, New South Wales; and 3) an Endocrine surgical database from the Royal North Shore Hospital in Sydney, New South Wales.

Eligible patients participating in the QTCS were invited by a research nurse from the QIMR Berghofer Medical Research Institute to participate in an additional interview about their thyroid cancer diagnosis. Interested patients were provided with additional information about the study and those who gave verbal consent to be contacted by the University of Sydney researchers were mailed a letter, Participant Information Sheet (PIS), and a formal consent form with a reply-paid envelop. Those who returned the consent form to researchers were called to arrange a mutually convenient time to conduct a telephone interview. All patients recruited via the QTCS were interviewed by telephone as patients lived throughout the state of Queensland in Australia.

Eligible patients presenting to patient clinics at the Chris O’Brien Lifehouse were recruited by two thyroid surgeons. Patients were given an Expression of Interest (EOI) form along with the study’s PIS. Interested patients then mailed the EOI form back to researchers in a reply-paid envelope and once researchers received patients’ EOI forms, they were contacted via telephone to set up either a face-to-face or telephone (if appropriate) interview. Consent forms were signed by the patients in person before the interview commenced or were mailed to patients if they were interviewed by telephone.

Recruitment from the Royal North Shore Hospital was conducted by a research database manager identifying patients from an Endocrine database of patients who had previously consented to be contacted for research purposes. Patients were identified if they met the eligibility criteria and were sent a letter, PIS and EOI form. Once researchers received the EOI form from interested patients, they contacted patients via telephone to set up either a face-to-face or telephone (if appropriate) interview. Consent forms were signed by the patients in person before the interview commenced or were mailed to patients if they were interviewed by telephone.

### The research team

Following the COREQ checklist for reporting qualitative research [12] the research team has expertise in public health, healthcare communication, endocrinology, general medicine, clinical epidemiology, psychology and overdiagnosis. BN is a PhD candidate and public health researcher specialising in communication and overdiagnosis. JPB is an international endocrinologist who specialises in thyroid disorders communication and decision making research. RM is a post-doctoral researcher with a special interest in overdiagnosis. AB is an epidemiologist and public health researcher with expertise in cancer screening and early detection. SJ is a cancer epidemiologist with special interests in the aetiology and diagnostic pathways for thyroid cancer. KM is a health psychologist and senior qualitative researcher with an interest in overdiagnosis and communication science.

### Data collection

The semi-structured interview schedule (see Additional file 1) was developed and revised with advice from an international endocrinologist who specialises in thyroid cancer communication and decision making research (JPB), as well as qualitative research (KM) and public health (AB) experts. The interviews were conducted between October 2015 and July 2016, and took place at patients’ homes or by telephone. The interviewer was a public health researcher with experience conducting qualitative interviews and using qualitative research methods (BN). Interviews lasted between 15 and 40 min and were audio-recorded and transcribed verbatim.

### Analysis

A thematic analysis of the transcribed interviews was conducted using Framework Analysis to organise the data to capture the views expressed by patients [13]. To ensure rigor in the analysis a step-by-step approach was taken by the research team [14]. The first step was familiarisation of the data, where one researcher independently reviewed and made notes on all 25 interview transcripts. Next was the creation of a thematic framework where 3 researchers (BN, JB, KM) read a sub-set of transcripts covering a range of ages and genders, and developed and revised with continuous discussions the framework based on emerging topics and themes arising from the transcripts. Following the development of the framework, an additional researcher (RM) read a further sub-set of interviews and approved the framework for coding. Coding was done by two researchers (BN and RM), where BN independently coded all 25 interviews into the framework with RM independently double-coding a random set of 3 interviews. Similarities or differences in the coding between the two researchers was discussed and re-assessed. Once coding was complete, BN and RM examined the framework within and across themes and participants to identify the overarching themes and relationships. These themes were summarised and checked by two additional members of the research team (JB and KM) by each reading an additional transcript. The final results were further discussed with the entire research team.

## Results

Participants were 17 women and 8 men, between 23 and 78 years of age, from a range of demographic backgrounds and levels of education (see Table 1).

**Table 1:** Participant characteristics

Characteristic	No. of patients (*n* = 25)
Age (years)	
≤25 26–50 51–75 75+	1 5 18 1
Gender	
Female Male	17 8
Highest level of education	
Year 11 or below High school Technical or college diploma Bachelors degree or above	2 2 8 13
Recruitment Avenue	
Queensland Thyroid Cancer Study (QTCS) Chris O’Brien Lifehouse patient rooms Royal North Shore Hospital endocrine surgical database	18 2 5
Size of nodule (mm)^a^	
0.0–1.9 2.0–3.9 4.0–5.9 6.0–7.9 8.0- ≤ 10.0	3 3 6 6 5
Mode of detection^b^	
Physical exam finding Imaging finding Diagnostic work-up for thyroid abnormality Case finding Symptomatic nodule	3 14 3 1 4

^a^Patient reported, two patients were unsure of the exact size of their nodules. All nodules were ≤10 mm

^b^How the patient came to be diagnosed with PMC from information discussed during the interview based on Singh Ospina’s definition of mechanism detection [15]

From the thematic analysis, study findings were organised into six main areas to illustrate the themes arising from patient reports of their experiences and their views expressed during the interviews: the diagnostic pathway; understanding of information related to a PMC diagnosis; treatment decision making; impact of being diagnosed and receiving treatment; terminology and the effect of the word cancer; and feeling towards active surveillance.

### The diagnostic pathway

Patients reported experiencing different pathways that ultimately led to their diagnosis. Based on Singh Ospina’s mechanisms of detection definitions [15], most patients were asymptomatic and their PMC diagnosis was initiated by an incidental finding during imaging tests (e.g. CT scan for a pinched nerve). Commonly, general practitioners (GPs) were the first point of contact and sent patients for imaging tests or referring patients to thyroid specialists.

Other patients, whose PMC was not detected during imaging tests, had their nodules initially identified by either a physical examination (palpation of the neck in an asymptomatic patient); diagnostic work up for symptoms suggestive of thyroid hormone disorder; case finding (thyroid cancer screening in an asymptomatic patient who may have had exposure to nuclear fallout from Chernobyl); and work up for a symptomatic nodule (symptoms present e.g. feeling or seeing a lump). These mechanisms of detection were less common than detection during an imaging test. See Table 2 for classification of modes of detection and patient examples.

**Table 2:** Classification of modes of detection for PMC with patient examples and supporting quotes

Classification^a^	Description	Patient example	Supporting quote
*Asymptomatic nodule*			
Physical exam finding	When a thyroid nodule is found during a physical exam (thyroid palpation) of a patient without symptoms concerning for thyroid disorder	Patient went to a local GP for a check-up and the GP felt a lump on his throat	*“I went to the local doctors just to sign up, and not that there was anything wrong with us, but, er, and while we were talking the doctor said to me, what’s that lump in your throat?”*
Imaging finding	When a thyroid nodule is found during an imaging test requested for reasons unrelated to a thyroid disorder or symptom	CT scan for a pinch nerve in patient’s shoulder	*“I was feeling, electrical pulse, pulses going through my shoulder, through my arms to my fingers. And, um, that happened for a few days and then I was worried and I went to see my GP. [He said] “… we’re going to do a scan you might have a pinched nerve or something.””*
Diagnostic work up	When a thyroid nodule is found during the work up of clinical symptoms or abnormal laboratory tests that could be suggestive of thyroid disease or known thyroid disease	Patient was not feeling well and blood tests revealed that something may be wrong in her thyroid	*“I just went to see the doctor to just see you know there might be something wrong with my chest. So, we did an x-ray and she said look it’s fine. Everything is cleared however we’ll just do a blood test to see if your immunity is maybe a bit low and that’s where she found that my thyroid level was a bit playing up. Which I’d never had any problems before so she just did another test just to make sure and that’s when I sort of, that’s when she sort of found out that there is a bit of a problem.”*
Case finding	When a thyroid nodule is found on an ultrasound performed for thyroid cancer screening	Patient lived in Belarus during Chernobyl therefore clinician requested she be screened	*“I’m originally from Belarus, and I’m living in Australia for the last 4 years and we have big impact of Chernobyl, what all my doctors think and the, I didn’t have any symptoms anything, I just visit, when I was in Belarus for holidays I just went for the check-up. And they said that because of all my friends had similar problems I thought I just go and check and they said I have a nodule and then I was returned to Australia and they started to keep an eye on it.”*
*Symptomatic nodule*	When a thyroid nodule is found in a symptomatic patient	Felt a lump in his neck so went to the GP to get it checked	*“I turned my head I felt strange and I felt in my throat and, er, there was a lump. And, um, which I’d never seen before. And it did come up quite suddenly and it was visible. Um, so, um, I checked with my GP who said it was my thyroid, and he referred me to a specialist.”*

^a^As classified and described by Singh Ospina et al. 2016 [15]

Patients discussed being unaware of the possibility that these imaging tests could pick up other conditions. In particular, no patient discussed or seemed to be aware of the possibility that thyroid cancer could be identified.

*“I was just shocked. Because I… because… I went in to the… I went in for a scan on the… for the lump on the back on my neck, and they… and you know, I came out… you know, with the diagnosis of cancer.”* (female, age 51–75, imaging finding)

Once a thyroid nodule was identified patients were directed to have an ultrasound guided fine needle aspiration biopsy (USFNA) to determine whether the nodule was malignant. In general patients were advised to have an USFNA without any discussion of whether there might be alternative options or what the consequences of having it might be. Overall, requesting a biopsy seemed to be an almost automatic recommendation from the clinician following identification of a nodule. A few patients did not even seem sure why a biopsy was needed in the first place.

*“Before I went to go get the biopsy I think I wasn’t really quite sure why. But then I kind of thought maybe it was because they didn’t want to scare me with the information if it wasn’t actually needed.”* (female, age ≤ 25, symptomatic nodule)

### Understanding of information related to a PMC diagnosis

Information regarding a diagnosis of PMC was communicated to patients by various clinicians. Overall, most patients suggested that the clinicians described the tumours as being a “small” or “slow growing cancer”, and patients reported being told that surgery would offer a cure.

*“Well actually what he [thyroid surgeon] said to me was, um, he said, “This is a good result” …Um, he said, “Because it’s so small and it’s encapsulated and we found it early and everything”. I said, “So what’s the ramifications for me?”, and he said, “Basically, once you remove it that should be end of story”.”* (male, age 51–75, imaging finding)

Patients did not however appear to understand the natural history and generally indolent nature of PMC. As such, they did not seem to be aware of the possibility of overdiagnosis and overtreatment. Some patients seemed to be conflicted by their cancer diagnosis, as they understood cancer to be something terrible but they were told and felt that their cancer was a ‘good result’.

*“I understood that I had, obviously that I had cancer and to me I just wanted it out, I wanted to be able to move on with my life and just get it out and let’s be done with this and let’s get it done as soon as possible.”* (female, age 26–50, imaging finding)

*“I think it’s [cancer] built its reputation for a reason I suppose, that’s the way I look at it, I mean, it’s not to be taken lightly and, it’s, um, you know, it needs to be, needs to be addressed as quick as you can and, um, you know, there’s obviously all, all grades in all types and what have you but, um, it’s all cancer, isn’t it?”* (male, age 51–75, imaging finding)

### Treatment decision making

For the vast majority of patients, they reported that surgery (thyroidectomy or hemi-thyroidectomy) was the only treatment option recommended or discussed; only one patient reported being offered the option of active surveillance. For some patients, total thyroidectomy was the only treatment suggested, while others were offered either a total or hemi-thyroidectomy. In some instances, the clinician offered both options but recommended a hemi-thyroidectomy to try and reduce possible adverse effects and medication burden for the patient. Most patients described having discussions with their clinicians about the risks and side-effects associated with surgery.

*“…because they knew it was there they said they had to take it out*.” (female, age 51–75, imaging finding)

*“So he didn’t, I don’t think there was any way to give me options, you know. The option was ok, we have to take it out. The only option.”* (male, age 51–75, imaging finding)

Overall, patients placed their trust in their clinician’s treatment recommendations and did not usually question their treatment advice.

*“Um, I didn’t ask the question why I need to have the whole thyroid removed at all, because I just felt they are the experts.”* (female, 51–75, imaging finding)

*“I just think I just agreed, um… just agreed to everything that was, that was happening. Um, I don’t think I had… enough time to really, um… digest it.”* (female, age 26–50, symptomatic nodule)

A number of patients did however describe their personal preference for treatment. Patients who preferred a total thyroidectomy discussed not feeling comfortable having something cancerous in their body and not wanting to deal with any further risks (including the possibility of needing further surgery) that may arise. These patients wanted to take a ‘better safe than sorry’ approach to their treatment. Patients who reported preferring a hemi-thyroidectomy discussed the importance of not wanting to be on thyroid replacement medication for the rest of their lives. These patients tended to place a high emphasis on the impact it may have on their lifestyle or had heard stories about the difficulties and side-effects people encounter on thyroid replacement medication.

*“I wanted everything out of my body. I was happy to have the thyroidectomy; I just wanted everything that was cancerous out.”* (female, 51–75, imaging finding)

*“I’ve heard horror stories… So I’m very wary of taking medication, because I’ve worked in doctor’s surgeries, I’ve seen so many people pop pills and not ask why. I come at that from a very different perspective.”* (female, age 51–75, imaging finding)

### Impact of being diagnosed and receiving treatment

Patients experience with physical, emotional or psychological side-effects varied. Some patients described almost no side-effects while others had mild to severe side-effects following their diagnosis and treatment of PMC. Fatigue was the most common physical side-effect that was discussed during the interviews. Many patients described having fatigue following their surgery and also, many patients who received a total thyroidectomy described having fatigue upon starting their thyroid replacement medication.

*“I’ve had so many times that I’ve felt so tired, I’ve felt pretty awful a lot of the time since the surgery.”* (female, age 51–75, imaging finding)

Changes in voice and issues with calcium levels following surgery was also described by a few patients. Some of these patients described having more difficulty following surgery than other patients, as they had to stay in the hospital for longer periods than expected or have additional medication.

*“The thing is my voice is not the same. It’s not the same. Like I’m talking now, sometimes after days, waiting for days it’s a bit better, but every single day when I wake up in the morning my voice is, um… oh, what’s the word? My voice is croaky.”* (male, age 51–75, imaging finding)

*“I know they did, um… nic one of the parathyroids and since I had my thyroid, thyroid out, um… um, I’ve been just having a lot of trouble with my calcium levels trying to stabilise my calcium levels. Um, been in and out of hospital having, um, calcium infusions.”* (female, age 26–50, diagnostic work-up)

**Fig. 1:**
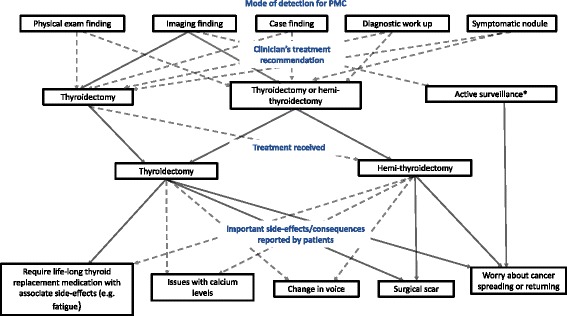
Treatment pathway and important side-effects/consequences of treatment reported by patients Legend: Solid line (−) = common, Dotted line (--) = less common, *only 1 patient offered active surveillance

In terms of emotional and psychological side-effects, a number of patients who received either a total thyroidectomy or a hemi-thyroidectomy discussed being continually worried that the cancer could spread or return. See Fig. 1 for treatment pathway and important side-effects/consequences of treatment reported by patients.

*“I worried all the time, you know, sometimes, even today, one year after. Even today sometimes I go to bed and sort of, er, thinking about ok, what’s wrong with my, er, voice and, and, er, am I going to get cancer on the other side, or is it going to spread, you know? I’ve got that worry in my mind all the time, yeah, there is no doubt.”* (male, age 51–75, imaging finding)

Generally, patients expressed feeling lucky that their cancer was caught early and that it was treated quickly.

*“I felt very lucky because of, um, it was, you know, a very early diagnosis really.”* (female, age 51–75, imaging finding)

### Terminology and the effect of the cancer term

When patients were asked about different terminology to describe PMC, which has been proposed by a number of cancer experts and researchers [4, 16], they generally seemed unfamiliar with terms other than cancer to describe their condition. Moreover, patients could not describe how they might react if their diagnosis called something other than cancer.

Patients did however describe their experiences when hearing the word. The effect of being diagnosed with cancer varied across patients. Many patients described being in complete shock as they were not expecting a cancer diagnosis. A few of these patients described feeling panicked and thinking that cancer meant that were going to die.

*“It’s like a, it’s like a freakin’ tsunami…. I think that “C” word you don’t… you can prepare for it, but you can never really... understand until you’ve been told, “You have cancer.””* (male, age 51–75, symptomatic nodule)

*“Well they say it was cancer, I tell you what, I’m thinking ok, I’m going to die. Whatever they tell me, ok, I’m going to die soon, I’m going to die.”* (male, age 51–75, imaging finding)

On the other hand, a few patients described not feeling too worried about their diagnosis.

*“...if you’re going to have a tumour perhaps that’s not a bad place to have it.”* (female, age 51–75, imaging finding)

### Feelings towards active surveillance

When asked how they would feel, retrospectively, about active surveillance as a potential management option, few patients were open to this idea. As mentioned previously most patients did not like the idea of having a cancer inside their body and wanted it removed quickly.

*“I just want to get in and get it fixed, I just think with any type of cancer; I don’t think they should be sitting in your body. Whether they are slow growing or not I just don’t think they should be there. And if there is a way, if it was my body and my choice I want it out as soon as possible.”* (female, age 51–75, imagingl finding)

However, one patient did suggest he would have liked to have more information about active surveillance and when directly asked said he may have chosen it if it was offered to him at the time.

*“I would’ve also liked, um, somebody to be a bit frank about, um, about the controversy. You know, that, that... There is a way to follow it up by monitoring.”* (male, age 51–75, imaging finding)

## Discussion

In a sample of Australian patients diagnosed with PMC, the diagnosis and treatment experience varied widely. The majority of patient’s PMC were asymptomatic and initially detected via an imaging test requested for a reason unrelated to a thyroid disorder or symptom. This incidental finding of a thyroid nodule led patients down a diagnostic and treatment cascade, which often resulted in physical and emotional side-effects.

GPs, who were the first point of contact for patients, generally did not seem to inform patients about possible consequences of further testing, as many patients described being surprised by their cancer diagnosis and unaware of the possibility that a cancer could be identified from the imagining test.

Clinicians (thyroid surgeons or endocrinologists) generally described PMC to the patients as being a “small” or “slow-growing” cancer. There was little evidence that clinicians had discussed the possibility of overdiagnosis and overtreatment of PMCs with patients. Patients understood that surgery offered them a cure, but did not seem to be aware that they might not have needed to be cured in the first place. According to their reports, surgery was the only management option discussed and offered to the majority of patients. Only one clinician discussed the option of active surveillance with their patient. When deciding about the extent of surgery, patients who preferred a total thyroidectomy valued the possibility of reoperation and wanted to get it all removed at once. Patients who preferred to receive a hemi-thyroidectomy discussed not wanting to be on thyroid replacement medication for the rest of their life as the main reason, although seemed unware that they may still have a 1 in 3 chance of requiring thyroid replacement medication [17, 18].

When patients were asked about different terminology to describe PMC, they generally seemed unfamiliar with terms other than cancer to describe their condition and could not describe how they might react if their diagnosis called something other than cancer. However, as expected, hearing the cancer term had a major impact on some patients. These patients described having ongoing anxiety about their diagnosis and the idea that their cancer may return. On the other hand, some patients described being unworried about their diagnosis and felt that thyroid cancer was a ‘good’ cancer if they were going to get one. Importantly, most patients also felt that active surveillance is not something that they would be interested in for their diagnosis. These patients described not wanting to have a cancer left inside of them and had a preference for it to be removed quickly.

### Implications for research and practice

This study provides insight into PMC patients experience surrounding diagnosis and treatment communication and decision making, which may help understand potential drivers of thyroid cancer overdiagnosis and the implications for non-surgical management options or changes in terminology.

Firstly, these findings underscore a need for more information for health professionals and the public on the meaning of incidental diagnoses and the possibility of overdiagnosis, both generically and specifically for thyroid cancer.

Avoiding ultrasound guided-biopsy for thyroid nodules < 1 cm would reduce unnecessary detection of PMC. Guidelines have advised against biopsy of these small nodules for a number of years [19] and although there has been a reported shift in practice [20], as demonstrated by the patient experiences in this study nodules < 1 cm are still being biopsied. Providing information to patients and involving them in the shared decision making process, when appropriate, about whether or not to proceed with the biopsy may be an effective strategy to reduce unnecessary PMC diagnoses. Providing patients with decision aids may be one effective strategy [21, 22].

These interviews also showed that patients felt lucky to be diagnosed early and felt that their life had been saved by treatment, although several were still burdened with ongoing physical and emotional side-effects. PMC patients in this study provide an example of how overdiagnosis can create a cycle of positive feedback for more overdiagnosis, as described by Welch and Black [23]. As thyroid cancer incidence increases so does population awareness (e.g. more patients might hear about someone they know who has been diagnosed with thyroid cancer and saved). This increased awareness, combined with the general enthusiasm for early detection, may lead some people to be diagnosed and receive treatments that they feel may save their life. For thyroid cancer, the problem is also compounded by high survival rates that many may solely attribute to surgical interventions.

Reducing the number of PMC diagnoses is the best strategy to stop the overdiagnosis cycle; however, if a patient does receive a diagnosis it is important that clinicians give a priority to shared decision making when discussing management options. Evidence suggests that this should now include discussing the option of active surveillance with those diagnosed with PMC [24]. Further research is required to fully understand how a change in terminology for PMC would impact patients’ treatment decision making and psychosocial outcomes as it has been shown for other lesions with low malignant potential (e.g. ductal carcinoma in situ) that removing the term ‘cancer’ from the diagnosis may help alleviate anxiety, allowing patients to give greater consideration to more conservative management options [25, 26].

### Strengths

This is the first qualitative study which explores the diagnosis and treatment experiences of patients with small low-risk thyroid cancers. Understanding communication, decision making and treatment preference surrounding a diagnosis of PMC is important and timely as evidence surrounding potential overdiagnosis of these lesions is emerging and management options are changing. We specifically only interviewed patients who had been diagnosed pre-operatively with PMC < 1 cm as these smaller thyroid tumours are thought to be important drivers of increasing incidence rates [1], and at this time, this is the group of patients where active surveillance can be considered as an alternative management option to immediate surgery [10]. The age and sex distribution of patients interviewed in this study reflects what is expected in a thyroid cancer population [1, 5], however there was a greater proportion of patients with higher levels of educational attainments which is likely reflective of higher educated people being more willing to take part in research.

We used rigorous qualitative methods which included recruitment of patients from a number of avenues to ensure there was a heterogeneous sample of patient experiences, theme saturation, a trained and experienced qualitative interviewer and a detailed analysis process to reach the final themes [14].

### Limitations

This study may be limited by the retrospective nature of the interviews. Interviewing patients during or directly after their diagnosis and treatment may have revealed more thorough accounts of the patient’s experience including the immediate physical and emotional impact. However, interviewing patients up to 1 year post-diagnosis and treatment did allow for patients to reflect on their experiences. Furthermore, a few of the patients interviewed were only a few weeks or months after they had completed their treatment and many patients were able to discuss their ongoing side-effects or struggles from their diagnosis and treatment experience.

Also, we only interviewed patients from Australia and therefore the experiences of PMC patients in other countries may differ. For example, in Japan where clinicians have more experience offering active surveillance for the management of PMC, patients may be more familiar and receptive to this option [9, 11]. As all patients in our study had already received surgery for their diagnosis of PMC, attitudes towards active surveillance were difficult to adequately measure or understand as they were subjected to biases such as cognitive dissonance.

## Conclusions

Evidence continues to emerge that PMCs are being overdiagnosed, and management guidelines are now recommending more conservative management options for these lesions. As a result, communicating these issues to patients with a PMC diagnosis is extremely important and shared decision making around treatment options is vital. This will help ensure that patients are fully aware of the meaning of their diagnosis, their management options, including active surveillance, and the associated benefits and risks. More importantly, interventions to reduce unnecessary diagnoses of PMC are critically needed. Reducing excessive imaging tests and educating patients about their choices before having a biopsy will likely reduce unnecessary physical and psychological harms associated with overtreatment of this indolent condition.

## Additional file

Additional file 1: Patient Interview Schedule. Semi-structured patient interview schedule. (DOCX 109 kb)

## Abbreviations

CT: computed tomography
GP: general practitioner
PMC: Papillary Microcarcinoma
PTC: Papillary Thyroid Cancer
