# Supplementary material for: Patients’ experiences of diagnosis and management of papillary thyroid microcarcinoma: a qualitative study
Source: BMC Cancer. 2018 Mar 2;18:242. doi: 10.1186/s12885-018-4152-9 (PMC5833084; doi:10.1186/s12885-018-4152-9)
Supplement: Supplementary file 1 — Patient Interview Schedule. Semi-structured patient interview schedule. (DOCX 109 kb) [file 12885_2018_4152_MOESM1_ESM.docx]

**Additional file 1. Patient Interview Schedule**

**Background of diagnosis**

1. Can you just take me through from the start how you became diagnosed with thyroid cancer?

Prompts:

- What took you to the doctor in the first place? What were your main symptoms or concerns?
- What specific tests were given to lead to the diagnosis? (Ie. CT, MRI, ultrasound guided biopsy)
  - Was the test and possible outcomes of the test discussed and explained to you before you received it?
- What was the exact diagnosis (name) that was given to you?
- What treatments were you ultimately given? Thyroidectomy/hemi-thyroidectomy/ + radioactive iodine therapy (RAI)?

**Diagnosis specifics *(if not mentioned or discussed previously)***

1. How was the diagnosis you were given explained to you? What were the medical terms that were used? Was this clear?
2. Who gave you the diagnosis (what type of doctor)? Were there multiple doctors? Did you consult another doctor about your thyroid cancer?
3. What was your understanding of the diagnosis?
4. How did you feel when you received the diagnosis? What were your initial thoughts? What are your thoughts now?

**Treatment decisions**

1. If we could just go back to the treatment you received. Can you just take me through your treatment and how the process to receive ____________________ *[type of treatment they received – thyroidectomy, hemi-thyroidectomy, + radioactive iodine therapy (RAI)]* began and how you feel/felt about your treatment and treatment process in general?

Prompts:

- What was the information that was provided to you about your treatment options?
- How was the treatment decided?
- What factors contributed to the decision making regarding the extension of your treatment (thyroidectomy vs. hemi-thyroidectomy)?
  - What was the doctor’s advice?
- Were you told about any risks associated with your treatment?
- How did you feel about the treatment you were going to receive?
- How prepared did you feel for the treatment process and life after?
- What were your expectations vs. your experiences of the treatment?

**Experiences after treatment/during recovery**

1. Now that you have had your treatment how are you feeling and what have your experiences since diagnosis and treatment been like for you?

Prompts:

- What have your physical experiences been like?
- How has your medication experiences been? – Any interactions? How about the costs associated with taking the medications?
- What has the follow-up plan been like? Doctors appointments? Blood tests? Any imaging?
- Have you had any problems psychological/emotionally since?
- What lifestyle changes have you endured?
- How have you found readjusting to your life since?

1. Is there any information that you would have like to have known that you weren’t told about?
2. Are there any aspects of your care that you think could have been improved?

***If not mentioned or discussed previously:***

1. How did you feel when you heard the word “cancer” during your diagnosis?”
   1. Do you think calling your diagnosis a different name or removing the word cancer would have made any of your feelings to the diagnosis or decisions with treatment change?

***If not mentioned or discussed previously:***

1. How do you think you would feel if your clinician proposed active surveillance for the management of your papillary thyroid cancer?
   1. Why do you think you would feel that way?
   2. What would give you confidence in this management option?

Prompt:

“Active surveillance is where doctors closely monitor the thyroid cancer nodule with ultrasound approximately once a year and only provide treatment if there is progression (for example tumour growth or lymph node metastasis)”
